# Proportion of unplanned pregnancies, their determinants and health outcomes of women delivering at a teaching hospital in Sri Lanka

**DOI:** 10.1186/s12884-020-03259-2

**Published:** 2020-11-05

**Authors:** Iddamalgoda Dissanayakage Jayani Chalindra Ranatunga, Kapila Jayaratne

**Affiliations:** 1 Postgraduate Institute of Medicine, University of Colombo, 160, Prof. NandadasaKodagoda Rd, Colombo, Sri Lanka; 2 Family Health Bureau, Ministry of Health, 231 De Saram Place, Colombo 10, Sri Lanka

**Keywords:** Unplanned pregnancy, Maternal and newborn health outcomes

## Abstract

**Background:**

Unplanned pregnancy is a significant public health issue in both low- and high-income countries. The burden of unplanned pregnancy is reflected in women opting for pregnancy terminations and it can be detrimental to the women and her family as well as the health system and society. Solid data on the proportion of unplanned pregnancies are using more specific tools such as the London Measure of Unplanned Pregnancy (LMUP) needed to address the issue in Sri Lankan contexts. The objective was to describe the proportion of unplanned pregnancies, their determinants and the health outcomes of women delivering at Colombo North Teaching Hospital-Ragama (CNTH).

**Methods:**

A cross-sectional study was carried out among 494 consecutive pregnant women selected by non-probability consecutive sampling who were admitted for the confinement at CNTH. A pre-tested structured interviewer-administered questionnaire was used to collect data on antenatal women and intentionality measured by self-administered six-item LMUP. Maternal and newborn health outcomes were ascertained in each post-partum women before discharge. Data were analyzed with the Mann-Whitney U tests, Kruskal-Wallis tests and spearman rank correlation. We also evaluated the psychometric properties of the Sinhalese version of LMUP.

**Results:**

The response rate was 97.8 and 17.2% of pregnancies ending at birth were unplanned, 12.7% were ambivalent and 70.1% were planned.

Associated factor profile of women with unplanned pregnancies includes; not married women (*p* = 0.001), educated up to the passing of GCE ordinary level by women (*p* <  0.001) and spouse (*p* <  0.001), primiparity (*p* = 0.002) and inadequate knowledge on emergency contraceptives (*p* = 0.037). Less planned pregnancies were also significantly associated with anemia (*p* = 0.004), low mood for last 2 weeks (*p* <  0.001), having a partner with problematic alcohol consumption (*p* <  0.001), presence of Gender-Based Violence (GBV) (*p* < 0.001), poor relationship satisfaction with partner (*p* < 0.001) and family (*p* < 0.001). Inadequate pre-pregnancy preparation and antenatal care were associated with an unplanned pregnancy. No differences were found in neonatal outcomes. Sinhalese version of the LMUP scale was found to be accepted, valid and reliable with the Cronbach’s alpha of 0.936.

**Conclusions:**

A sizeable proportion of pregnancies were unplanned. Teenage pregnancies, non-marital relationships and inadequate knowledge on emergency contraceptives, maternal anemia, low mood, and GBV were modifiable associated factors which could be prevented by evidence-based locally applicable approaches.

## Background

Unplanned pregnancy is a pregnancy that is either mistimed (pregnancy occurring earlier than desired) or unwanted (when no children or no more children are desired) at the time of conception. A planned pregnancy occurs at the desired time or later [1]. Women have more pregnancies than the number of children they want and tried to suppress such pregnancies using contraception to bring out wanted off springs [2].

Estimates indicate that 213 million annual pregnancies occur worldwide. Of these, the majority (56%), occurred in Asian countries [2]. In Sri Lanka 334,821 live births were reported in 2016 [3]. Of the global pregnancies, 40% were unplanned [2], of which 50% ended in abortion, 13% were miscarriages and 38% were live births [2, 4]. In low and middle-income countries, the incidence of unplanned pregnancies varies from 14 to 62% with Nepal having 41% [5], Pakistan 38.2% [6], Bangladesh 30.3% [7] and Sri Lanka 23.3% [8].

Unplanned pregnancies are also termed unintended pregnancies. Unintended pregnancy is a fundamental concept that is used to explain the fertility of populations and the unmet need for family planning. Such pregnancies mainly result from poor knowledge on contraception [9], non – use of contraception [10] or inconsistent or incorrect use of effective contraceptive methods [11, 12]. This may also invariably be due to an earlier age of starting sexual life and the increasingly popular concept of small family size [13]. Other potentially associated factors are teenage pregnancy [14, 15], first pregnancy as well as high parity [16], low educational status of the mother or father [17], poverty, lack of social support, Gender Based Violence (GBV), physical and sexual violence [18] and low birth interval [6].

Women with unplanned pregnancy present later for the antenatal care and attend less antenatal clinic visits [19, 20]. Women with unplanned pregnancy are more prone to get suboptimal nutritional supplements including peri-conceptional folic acids, micronutrients with having more unhealthy behavior during pregnancy and maternal smoking [21]. Available evidences show that unplanned pregnancies can have multiple negative maternal outcomes than those that are planned. Many women with unplanned pregnancies have hyperemesis gravidarum, bladder or urinary infection [16], feeling unwanted about the current pregnancy, postpartum stress, and depression [22, 23], unsafe abortion [24, 25], an increased risk of obstetric complications such as obstructed labor, anemia, and preeclampsia which can result in long-term morbidities [26]. Unplanned pregnancy is a significant public health concern that predispose to women to maternal deaths mainly through poor maternity care and unsafe pregnancy terminations [27]. The newborn is also not spared of negative consequences in unplanned pregnancies with higher incidences of low birth weight, prematurity [28], stillbirth, neonatal mortality [29], cognitive delay, substance abuse [30] and behavioral problems [31]. These pregnancies continue to be a burden to society, health systems and economies of countries [32].

The cost of an unplanned pregnancy is high because the woman has an option of either carrying the pregnancy to term and retaining the baby [33], decide on the adaptation of baby or to have an induced abortion [34, 35], even in settings where termination of pregnancy is not legal [36]. Adverse physical and psychological effects on mothers, newborns, and families initiate a cascade of consequences that could run through several generations.

The consequences of negative health, economic, social and psychological outcomes for women and children are associated with unplanned pregnancies and thereby making it a high priority area in health agendas [2, 11].

The reduction of unplanned pregnancy is a key concept in the Global sustainable development agenda in 2030. The global vision is that every woman will celebrate a wanted, healthy pregnancy, and safe birth of a child who will not just survive but thrive to his or her full potential [37, 38].

Sri Lanka is a lower-middle-income country in South Asia where ranges of reproductive health services were declared to be free for all people regardless of their ability to pay. Pre pregnancy care, family planning services, antenatal care, skilled delivery, basic and comprehensive obstetric and newborn care are given by primary care facilities and government hospitals. Medical officer of health, Public health nurses and midwives responsible for the family planning services at the grass root level [39].

According to 2016 Demographic and Health Survey (DHS), it has been revealed that the awareness of family planning in Sri Lanka was 98%, contraceptive prevalence was 70%, prevalence of modern methods was 53% and unmet need 7% [40]. Pregnancy terminations are illegal according to country law other than for the life-saving purpose of the mother. Abortion is a common method of fertility control and more than 500 abortions are done daily in Sri Lanka [41]. The majority is undertaken in unsafe conditions leading to complications and adverse health outcomes [42, 43].

The national maternal death surveillance and response system has also revealed that the index pregnancy was unplanned and unwanted among 39% mothers who died from 2001 to 2005 and 18% unmet need among maternal deaths in the year 2016 [44].

The reported prevalence of unplanned pregnancies in Sri Lanka varies from 23 to 46% [8, 45]. A community-based cross-sectional study conducted in 2010 in the Bentota MOH area revealed that 46.7% unplanned pregnancies [45]. A study conducted in the Colombo Municipal Council in 2015 revealed 44% unplanned pregnancies [46] and a study in the General Hospital Matara in 2013 revealed 23.3% unplanned pregnancies [8].

Proportion estimates of unplanned pregnancy were based on a single question with the dichotomous variable in most studies in Sri Lanka. Most studies were focused on unplanned pregnancy as a subsection of another research objective. These measures are not sufficient to measure the burden of unplanned pregnancy accurately. Furthermore, LMUP is a validated tool that has been widely used in many countries and is quick to complete. The six-item Sinhalese LMUP version was used for the estimation of the proportion of unplanned pregnancy in this study. Although extensive research has been carried out on the prevalence and associated socio-demographic profile of unplanned pregnancies, limited research data were available on maternal and newborn health outcomes. To the authors’ knowledge, this is one of the first studies to investigate the prevalence, associated factors and health outcomes of women who continue their pregnancy until delivery in Sri Lanka.

The purpose of conducting this study was to describe the proportion of unplanned pregnancies using LMUP and investigate the associated factors and health outcomes of pregnancies ending in birth at the Colombo North Teaching Hospital, Ragama (CNTH).

## Methods

### Study setting and participants

A hospital-based cross-sectional study was conducted from August to October 2017 in the CNTH-Ragama, Sri Lanka. The hospital-based study provides an opportunity to gather information from almost all women residing in this catchment area as nearly 100% of deliveries take place in hospitals in Sri Lanka. The CNTH is the premier hospital in the Gampaha District situated in the Western Province in Sri Lanka. This hospital extends across 27 acres of land and receives a heterogeneous mixture of patients representing different ethnic and religious groups. The study was carried out in all three obstetrics and gynecology units in the CNTH which had an average of 500 deliveries per month.

The sample size was calculated using the prevalence rate of unplanned pregnancies of 23.3% [8], a confidence interval of 95%, a precision of 4% and a non-response rate of 15% [47]. The total computed sample size was 505.

The study was conducted on consecutive women who were admitted for confinement to the CNTH. The study subject recruitment was based on the admission register and all the pregnant women who were admitted for delivery or women with signs of labour were included irrespective of age and the period of gestation (POA).

Pregnant women were in emergency care, sever pain, sick and women who admitted for reasons other than confinement were excluded. Women who were unable to read or write English, Sinhala or Tamil were also excluded.

### Study instruments

The development of the questionnaire was done by an extensive literature review of pre-existing studies. The questionnaire was reviewed by a panel of four experts who included the Consultant Obstetrician & Gynecologist, the Consultant Paediatrician, the National Programme Manager of Family Planning and the Consultant Community Physician to assess the content validity of the instrument. The questionnaire comprised three sections; section one was the interviewer-administered questionnaire which consisted of the socio-demographic profile, family planning methods, psychosocial risk factors, and antenatal characteristics. Section two contained the self-administered Sinhalese or Tamil version of the LMUP to ascertain the intentionality of the current pregnancy and section three contained maternal and newborn data extraction forms.

Section one: Socio-demographic profile consists of ethnicity, religion, the highest educational level and state of employment of women and spouse, monthly income, marital status, age at marriage and age at conception. Age at marriage was used instead of age at first sexual intercourse because of its sensitive nature and attendant cultural beliefs.

Family planning characteristics of study participants were determined by the most recent family planning method used, reasons for not using any contraceptive methods and awareness on emergency family planning methods.

Psychosocial risk factors at the time women became pregnant were obtained by women recall data. Psychosocial risk factors obtained from the current antenatal package in Sri Lanka (Consultant community physicians, personal communication). Presence of current or past history of mental illness, deliberate self-harm, psychoactive substances or alcohol use, low mood during the last 2 weeks, lack of interest in usual pleasurable activities, experience of any stressful life event during the last 6 months, inability to carry out daily functions, inability to care for the baby, presence of financial issues, having physical or psychological handicapped children at home, problem drinking/substance abuse in partner, GBV, poor relationship with husband and poor relationships with family members were included for psychosocial risk factors. Antenatal characteristics were assessed by the behavior during pregnancy, preparation and the number of clinic visits.

Section two: Pregnancy planning state was evaluated using the Sinhalese and Tamil version of the self-administered LMUP. The LMUP consists of six sections covering contraceptive use, pre-conceptional preparation, circumstances and timing, desire for pregnancy and motherhood, partner influence and intention to become pregnant. Each section scored 0 to 2 with a total score ranging from 0 to 12 [48, 49]. Prevalence or proportion estimation, LMUP score was divided into three groups: 0 to 3 (unplanned), 4 to 9 (ambivalent) and 10 to 12 (planned).

It was decided to measure the intentionality of the pregnancy before the delivery of the baby to obtain a more reliable response than after delivery. In a post-partum mother with the newborn, it is insensitive and unethical to ask the wantedness of the current baby. The mother’s response to planning state may be influenced by the nature of the delivery experience. Complicated delivery or post-partum period may negatively influence on the planning state.

Section three: Intra-partum and post-partum health outcomes, birth weight, maturity, breastfeeding adequacy, neonatal complication during hospital stay were obtained from Bed Head Tickets (BHT) and medical records (Additional file 1).

Pre-testing of the questionnaire was carried out among randomly selected pregnant women awaiting delivery at the Castle Street Hospital for Women in Colombo.

### Data collection

Principal investigator (PI) and a properly trained female fourth-year medical student who was fluent in both Tamil and English language collected data. All three antenatal wards were daily visited during the data collection period. Eligible women were selected according to the admission register and an information sheet (Additional file 2) was given to read. Informed written consent was obtained from the participants after explaining the purpose of the study, participation process, advantages, and disadvantages of participation (Additional file 3). A self-administered questionnaire that consists of LMUP was given at the bedside, once they completed the LMUP, the interviewer-administered questionnaire was delivered maintaining optimum privacy in the absence of any other. BHT number and admission data were documented in each questionnaire to trace the post-partum mother to avoid loss in follow up and duplication.

Intrapartum, neonatal and maternal health outcome details were extracted from the BHT, Mothers Card, Pregnancy Record, and relevant medical records before discharge in the post-natal wards. Newborn birth weight was measured by properly calibrated neonatal beam balance and the Head Circumference (HC) was measured using new measuring tapes from the same manufacturer. Written guidelines were given to standardize the measuring procedure and midwives were competent in the measurement of weight and HC. Information of the mothers who were early discharged prior to the second capture, left against medical advice, transfer out to other units or untraceable were obtained from BHT, birth registers in labour room or post-natal ward. All the questionnaires were assessed for completeness and accuracy at the end of each day. All the eligible consented pregnant women recruited based on the admission register until the total sample size was achieved.

Ethical approval for the study was obtained from the Ethical Review Committee of the Faculty of Medicine, University of Kelaniya(Ref No: 193/07/2017).

### Data analysis

Statistical Package for Social Science (SPSS) version 21 was used for data analysis. Questions were pre corded and operationalized prior to data entry and analysis. Descriptive analysis was done by frequencies or percentages for categorical variables and means for continuous variables. The proportion of unplanned pregnancy was calculated from all completed six components of the LMUP. The authors were recommended the categorization of the score into three groups for prevalence estimation and continuous score for inferential statistics. A higher score is associated with a higher level of planned pregnancy while lower scores are associated with an unplanned pregnancy. LMUP considered as continuous variable, median and interquartile rage calculated. LMUP was not normally distributed and non-parametric tests were used. Mann-Whitney U tests and Kruskal-Wallis tests were used to compare the difference in LMUP across the categorical variables. Spearman rank correlation coefficient was performed to analyze the correlation between the LMUP score and continuous variables. *P*-value < 0.05 was considered statistically significant.

To validate the Sinhalese version of the scale we conducted a psychometric analysis of the Sinhala LMUP. Sinhalese translation was carried out by two native Sinhalese speakers who were bilingual experts and translated back into English. Internal consistency of the scale was assessed using Cronbach’s alpha statistic with the standard cutoff point of 0.7 [50]. Acceptability was assessed by missing data rates with low levels of missing data indicates greater acceptability. The distribution of the total score was captured to evaluate the targeting of the scale. To access the item discrimination, the item response option endorsement values were checked and < 80% endorsement was considered acceptable [49, 51–53]. We assessed the item-total correlations with a minimum correlation of 0.2 as accepted. The construct validity of the LMUP was evaluated using principal component analysis. The LMUP scale was considered valid if all the items were loaded into one component with an Eigenvalue larger than one [49, 53, 54].

## Results

The minimal sample size was 505 and completed data were available for 494 women. Out of study participants, eight women failed to follow up after the delivery and three questionnaires were incomplete. The response rate was 97.8%.

### Proportion of unplanned pregnancy

Majority of pregnancies (*n* = 346, 70.1%) were planned at the time of conception whereas the proportion of unplanned pregnancies were 17.2% (*n* = 85) and 12.7% (*n* = 63) were ambivalent.

**Fig. 1:**
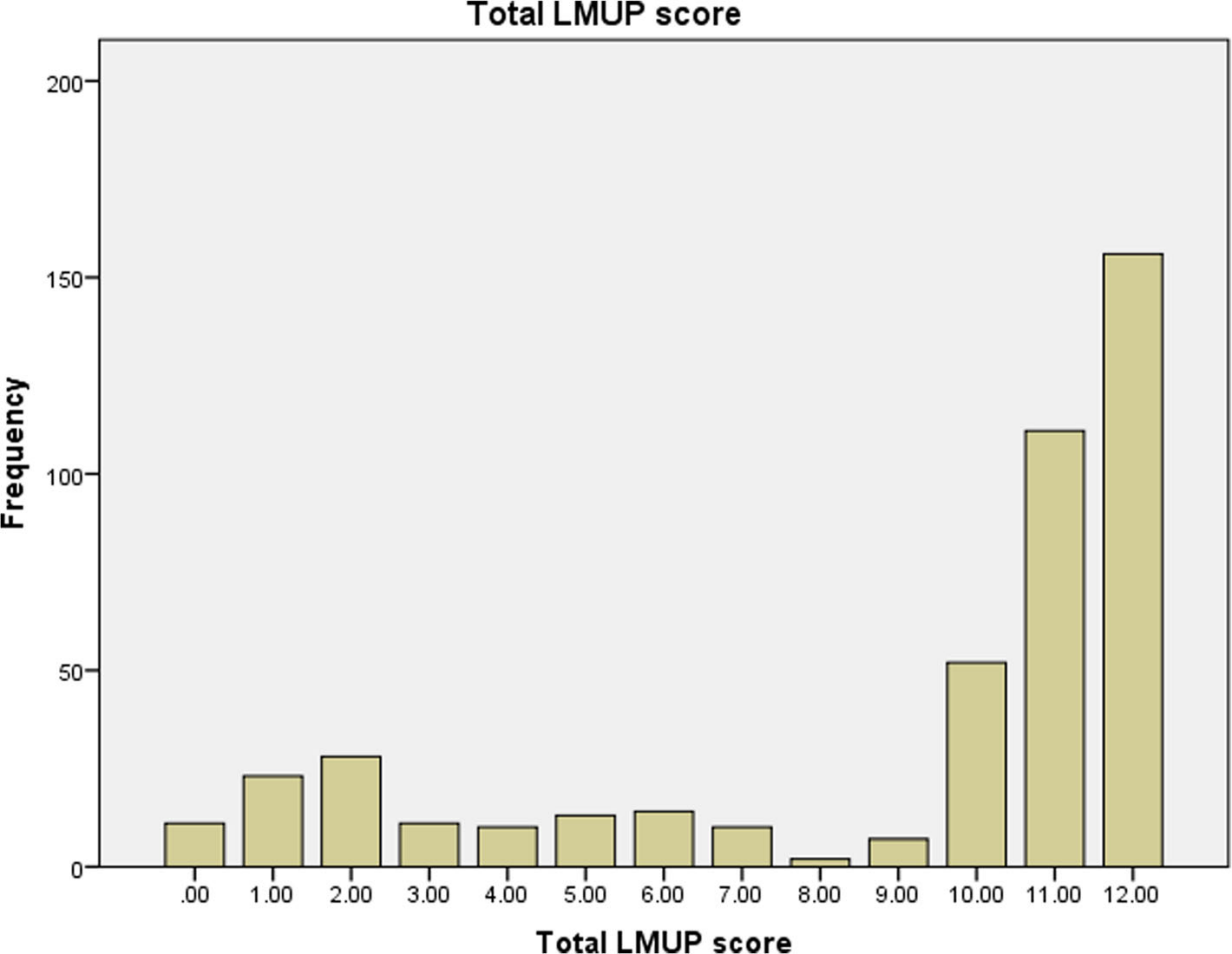
Frequency distribution of London measure of unplanned pregnancy

The mean LMUP score was 8.97 (95% CI 8.62–9.31) with a standard deviation (SD) of 3.914. Median was 11 and the interquartile range (IQR) was 6–12. Distribution was negatively skewed (− 1.135) (Fig. 1).

### Associated factor profile

The mean age of the study sample was 28.5 years (SD = 5.7), 90.6% were Sinhalese and 72.1% were Buddhist. The percentage of married women was 98.4%, mean age at marriage was 23 years and the average monthly income was Sri Lankan rupees 45,000 (1 USD = 179.68 LKR in 2016) [55]. When considering characteristics of women with unplanned pregnancy, majority (*n* = 79, 92.9%) were married, 51.7% (*n* = 44) married in their teens, 17.5% (*n* = 15) were teenage mothers. Sixty-one percent of women with an unplanned pregnancy and 83% (*n* = 70) of their husbands had been educated up to GCE Ordinary Level, 56.4% (*n* = 48) families had a monthly income less than LKR 40, 000.

Age at marriage (*p* < 0.001, r 0.22) and monthly income (*p* = 0.001, r 0.15) were both significantly and positively correlated to the LMUP score while non-marital relationship (*p* = 0.001), lower educational levels of both parents (*p* < 0.001) were significantly associated with an unplanned pregnancy. Socio-demographic characteristics are summarized in Table 1.

**Table 1:** Distribution of socio-demographic characteristics of the study participants

**Characteristics**	**Mean (SD)**	**Range**	**Spearman rank correlation test**	**Spearman rank correlation coefficient**
**Age at marriage (*in years)***	**23 (4.5)**	**14–39**	**< 0.001** ^**c**^	**0.222** ^**d**^
Maternal age at conception*(in years)*	28.5 (5.78)	15–48	0.331 ^c^	−0.44 ^**d**^
Monthly income (*LKR*)^*^	**45,000**	**8000–150,000**	**0.001** ^**c**^	**0.154** ^**d**^
**Characteristics**	**N (%)**	**LMUP score** ^**a**^	***p*** **value**	
**Ethnicity**				
Sinhalese	448 (90.6)	11 (7–12)	0.107 ^b^	
Non Sinhalese^e^	46 (41.3)	10 (3–12)		
**Religion**				
Buddhist	356 (72.1)	11 (7–12)	0.287 ^b^	
Other Religions^f^	138 (27.9)	11 (5.7–12)		
**Marital status**				
Not married	8 (1.6)	2 (1.8–5)	^*^ **0.001** ^**b**^	
Married	486 (98.4)	11 (7–12)		
**Educational level of women**				
Educated up to passing ordinary level	172 (34.8)	10 (2.2–11)	**< 0.001** ^**b**^	
Educated up to Grade 12 and above	322 (65.1)	11 (10–12)		
**Educational level of spouse**				
Educated up to passing ordinary level	292 (59.1)	10 (4–12)	**< 0.001** ^**b**^	
Educated up to Grade 12 and above	202 (40.8)	11 (10–12)		
**Employment status of women**				
Unemployed	364 (73.6)	11 (6–12)	0.664 ^b^	
Employed	130 (26.3)	11 (9–12)		

^*^1 USD =179.68 LKR in 2016

^a^Data are presented as median [interquartile range]

^b^Mann–Whitney U tests

^c^ *P* value of Spearman rank correlation test

^d^ Spearman rank correlation between the written characteristics and the LMU*P* value

^e^ Tamil, Muslim, and Burger were amalgamated

^f^ Catholic, Islam and Hindu were amalgamated

### Family planning characteristics

A larger proportion (76%, *n* = 65) of women with unplanned pregnancies were multiparous, 84.7% (*n* = 72) had never used any family planning method, of whom 70% (*n* = 51) were using modern methods) and 42.2% (*n* = 35) were not aware of emergency contraceptives. Associated factor profile of women with unplanned pregnancies included, multiparous (*p* = 0.002) women, who never used a family planning method (*p* < 0.001), practicing a modern family planning method (*p* = 0.04) and inadequate knowledge on emergency contraceptives (*p* = 0.037). Distance to the nearest family planning service provision station was significantly negatively correlated to the LMUP score (*p* = 0.03, r-0.09) (Table 2).

**Table 2:** Family planning characteristics in the study participants

**Characteristics**	**Mean (SD)**	**Range**	**Spearman rank correlation test**	**Spearman rank correlation coefficient**
**Distance to family planning service (km)**	**2.2 (2.0)**	**0.1–20**	**0.033** ^**c**^	**−0.096** ^**d**^
Birth Interval in years (*n* = 241)	4.85 (3.1)	1–17	0.484 ^c^	0.003 ^d^
**Characteristics**	**N (%)**	**LMUP Score** ^**a**^	***p*** **value**	
**Parity (*n*** **= 494)**				
Primiparity	199 (40.3)	11 (10–12)	**0.002** ^**b**^	
Multiparity	295 (59.7)	11 (4–12)		
**Miscarriages**				
No	395 (79.9)	11 (6–12)	0.237 ^b^	
Yes	99 (20.1)	11 (10–12)		
**Family planning precise** (***n*** **= 494)**				
Ever used	349 (70.6)	11 (5–12)	**< 0.001** ^**b**^	
Never used	145 (29.3)	11 (10–12)		
**Most recent practice** (***n*** **= 349)**				
Natural or traditional methods	143 (40.9)	11 (7–12)	**0.04** ^**b**^	
Modern methods	206 (59.1)	10 (3.7–12)		
**Modern method used (*n*** **= 206)**				
Oral contraceptive pills (OCP)	102 (49.5)	10 (3–12)	**0.04** ^**e**^	
Condoms	43 (20.8)	11 (4–12)		
Depot medoxy acetic acid (DMPA)	29 (14.7)	9 (2–11)		
Intrauterine contraceptive devices (IUCD)	25 (12.1)	11 (10–12)		
Implants	7 (3.4)	11 (6–11)		
**Knowledge on emergency contraceptives**				
Not adequate	265 (53.6)	11 (5.5–12)	**0.037** ^**b**^	
Adequate	229 (46.4)	11 (9–12)		

^a^ Data are presented as median [interquartile range]

^b^ Mann–Whitney U tests

^c^ *P* value of Spearman rank correlation test

^d^ Spearman rank correlation between the written characteristics and the LMU*P* value

^e^ Kruskal–Wallis tests

### Health of women and antenatal characteristics

Majority of unplanned pregnant women (55.2%, *n* = 47) did not consume peri-conceptional folic acid, 30% (*n* = 26) had visited less than four antenatal clinics and 44% (*n* = 38) had never attended any antenatal classes. The LMUP score significantly and positively correlated to the number of antenatal clinic visits (*p* < 0.001, r 0.30) and the number of antenatal classes attended (*p* < 0.001, r 0.36) while the period of amenorrhea (POA) at booking visit negatively correlated (*p* < 0.001, r-0.24). Delayed folic acid consumption (*p* < 0.001), presence of any medical conditions (*p*-0.018), maternal anemia (*p* = 0.004), preterm contractions (*p* = 0.042) were significantly associated with an unplanned pregnancy (Table 3).

**Table 3:** Health of women and antenatal characteristics of study participants

**Characteristics**	**Mean (SD)**	**Range**	**Spearman rank correlation test**	**Spearman rank correlation coefficient**
Pregnancy weight gain (Kg, *n* = 490)	9.552 (4.9)	1–39	0.093 ^c^	0.076 ^d^
POA at booking visit (weeks, *n* = 490)	**8.25 (4.3)**	**4–36**	**< 0.001** ^**c**^	**−0.249** ^**d**^
Number of antenatal clinic visits (*n* = 494)	**6.4 (1.9)**	**0–11**	**< 0.001** ^**c**^	**0.309** ^**d**^
Number of antenatal classes (*n* = 494)	**1.54 (1.1)**	**0–4**	**< 0.001** ^**c**^	**0.**369 ^d^
	**N (%)**	**LMUP score** ^**a**^	***p*** **value**	
**Folic acid consumption**				
After confirmation	206 (56.3)	6 (2–11)	**< 0.001** ^**b**^	
Peri or at confirmation*	288 (11.1)	12 (12–11)		
**Presence of medical conditions**				
Yes	245 (49.5)	11 (12–6)	**0.018** ^**b**^	
No	249 (50.4)	11 (6.5–12)		
**Hyperemesis gravidarum**				
Yes	21 (4.3)	10 (4.5–11)	0.27 ^b^	
No	473 (95.7)	11 (12–6)		
**Gestational diabetes**				
Yes	88 (17.8)	11 (8.3–12)	0.69 ^b^	
No	406 (82.2)	11 (6–12)		
**Antepartum hemorrhage**				
Yes	26 (5.3)	10.5 (5–11)	0.19 ^b^	
No	468 (94.7)	11 (6–12)		
**Pregnancy-induced hypertension (PIH)**				
Yes	41 (8.3)	11 (6–12)	0.67 ^b^	
No	453 (91.7)	11 (6–12)		
**Urinary tract infections**				
Yes	7 (1.4)	10 (6–10)	*0.39 ^b^	
No	487 (98.6)	11 (6–12)		
**Anemia**				
Yes	39 (7.9)	10 (3–11)	**0.004** ^**b**^	
No	455 (92.1)	11 (7–12)		
**Preterm contractions**				
Yes	16 - 3.2%	10 (3.5–11)	**0.042** ^**b**^	
No	478 - 96.8%	11 (6–12)		
**Prolonged rupture of membranes (PROM)**				
Yes	13 - 2.6%	11 (11–12)	0.62 ^b^	
No	481 - 97.4%	11 (6–12)		
**Other pregnancy problems/symptoms**				
Yes	49 - 9.9%	10 (6.5–11)	**0.012** ^**b**^	
No	445 - 90.1%	11 (6–12)		

^a^ Data are presented as median [interquartile range]

^b^ Mann–Whitney U tests

^c^ *P* value of Spearman rank correlation test

^d^ Spearman rank correlation between the written characteristics and the LMU*P* value

### Psychosocial risk during pregnancy

Lower LMUP score was significantly associated with the following psychosocial risk factors during the antenatal period. Women with a current or past history of deliberate self-harm (*p* = 0.009), presence of low mood during pregnancy (*p* < 0.001), women experiencing a stressful life event the during past 6 months (*p* < 0.001), women who perceived inability to carry out daily functions (*p* < 0.001), inability of care for the baby (*p* = 0.006), families with financial issues (*p* < 0.001)), having a partner with problem drinking (*p* < 0.001), women experiencing GBV (*p* < 0.001), poor relationship satisfaction with partner (*p* < 0.001) and poor relationship with family (*p* < 0.001) were associated with unplanned pregnancies (Table 4).

**Table 4:** Psychosocial risk factors of the study participants

Characteristics	N (%)	LMUP score ^**a**^	***P*** value
**Past history or current mental health**			
Yes	5 (1)	11 (9.5–11)	*0.85 ^b^
No	489 (99)	11 (6–12)	
**Past history or current Deliberate Self Harm**			
Yes	13 (2.6)	10 (2–11)	**0.009** ^**b**^
No	481 (97.3)	11 (6–12)	
**History or current use of Psychoactive substance**			
Yes	5 (1)	10 (3–10)	0.17 ^b^
No	489 (99)	11 (6–12)	
**Low mood during the past 2 weeks**			
Yes	77 (15.6)	10 (2–11)	**< 0.001** ^**b**^
No	417 (84.4)	11 (8.5–12)	
**Lack of interest in usual pleasurable activities**			
Yes	148 (29.9)	11 (8.3–11)	0.094 ^b^
No	346 (70.1)	11 (6–12)	
**Experience of any stressful life events in the last 6 months**			
Yes	37 (7.5)	5 (2–10)	**< 0.001** ^**b**^
No	457 (92.5)	11 (8–12)	
**Inability to carry out daily functions**			
Yes	163 (32.9)	10 (3–11)	**< 0.001** ^**b**^
No	331 (67.1)	11 (8–12)	
**Inability to care for the baby**			
Yes	157 (31.8)	11 (7–12)	**0.006** ^**b**^
No	337 (68.2)	11 (6–12)	
**Financial issues**			
Yes	48 (9.7)	6 (2–11)	**< 0.001** ^**b**^
No	446 (90.3)	11 (8.8–12)	
**Physical or psychological handicap children**			
Yes	8 (1.6)	11.5 (10.3–12)	0.25 ^b^
No	486 (98.4)	11 (6–12)	
**Problem drinking partner**			
Yes	117 (23.7)	10 (2–11)	**< 0.001** ^**b**^
No	377 (76.3)	11 (9–12)	
**Gender-Based Violence (GBV)**			
Yes	13 (2.6)	2 (2–8)	* < 0.001^**b**^
No	481 (97.4)	11 (7–12)	
**Relationship with the husband**			
Poor ^c^	43 (8.7)	6 (2–11)	**< 0.001** ^**b**^
Good ^d^	451 (91.3)	11 (7–12)	
**Relationship within family**			
Poor ^c^	55 (11.1)	10 (2–11)	**< 0.001** ^**b**^
Good ^d^	439 (88.9)	11 (7–12)	

^a^ Data are presented as median [interquartile range]

^b^ Mann–Whitney U tests

^c^ Poor, very poor answers were amalgamated

^d^ Average, good, very good answers were amalgamated

### Maternal health outcomes

Fifty-four percent (*n* = 46) of unplanned pregnant women had antenatal complications while only 5.8% (*n* = 5) had at least one post-partum complication. The majority of women with unplanned pregnancy 67% (*n* = 57) delivered vaginally and 44.7% (*n* = 38) had more than 3 days of hospital stay. None of them were associated with a lower LMUP score (Table 5).

**Table 5:** Intra-Partum, Post-Partum Maternal Outcomes

Characteristics	N (%)	LMUP Score ^**a**^	***P*** value
**Onset of Labour**			
Spontaneous	211 (42.7%)	11 (5–12)	0.35 ^c^
Induce	161 - 32.5%	11 (6–12)	
Planned caesarian section	122 - 24.8%	11 (7.7–12)	
**Fetal distress**			
Yes	44 (8.9)	11 (6–12)	0.46 ^b^
No	450 (91.1)	11 (6.7–12)	
**Mode of delivery**			
Vaginal	289 (58.5)	11 (5–12)	0.17 ^b^
Caesarian section	205 (41.5)	11 (10–12)	
**Presence of post-partum complications**			
Yes	41 (8.3)	11 (6–12)	0.86 ^b^
No	453 (91.7)	11 (6–12)	
**Post-partum hemorrhage**			
Yes	10 (2.0)	9 (2.7–12)	* 0.74 ^b^
No	484 (98.0)	11 (6–12)	
**Post-partum fever**			
Yes	12 (2.4)	11 (6.7–12)	* 0.53 ^b^
No	482 (97.6)	11 (6–12)	
**High Blood pressure**			
Yes	10 (2.0)	10 (5.3–11)	* 0.23 ^b^
No	484 (98.0)	11 (6–12)	
**Intensive care unit (ICU) stay**			
Yes	6 (1.2)	10 (5.3–12)	* 0.73 ^b^
No	488 (98.8)	11 (6–12)	
**Other complications**			
Yes	7 - 1.4%	12 (1–12)	0.71 ^b^
No	487 - 95.6%	11 (6–12)	

^a^ Data are presented as median [interquartile range]

^b^ Mann–Whitney U tests

^c^ Kruskal–Wallis tests

### Neonatal outcomes

There were ten multiple pregnancies and the 504 sample of newborns were included in the descriptive statistics. Multiple pregnancy per se has the potential to develop adverse newborn outcomes and therefore 20 newborns of multiple pregnancies were excluded from the analysis of association.

There were 16.4% (*n* = 83) preterm babies, 20.2% (*n* = 102) low birth weight babies and 22% (*n* = 107) had HC less than 2SD for their reference range. There were 88.6% (*n* = 429) babies had initiated breastfeeding within 1 h of delivery and only 3.1% (*n* = 15) had been fed with formula milk during their hospital stay. None of the above neonatal parameters were significantly associated with a lower LMUP score (Table 6).

**Table 6:** Neonatal Outcomes

**Outcome**	**Mean (SD)**	**Range**	**Spearman rank correlation test**	**Spearman rank correlation coefficient**
Birth weight (*grams*, *n* = 484)	2880 (572)	745–4750	0.689 ^c^	0.018 ^d^
Maturity (weeks, *n* = 484)	38 (2.21)	25–41	0.154 ^c^	0.064 ^d^
Head circumference (*cm*, *n* = 486)	32.6 (2.1)	21–44	0.067 ^c^	0.068 ^d^
Duration of hospital stay (*Days*)	4.3 (4.0)	2–65	0.752 ^c^	0.014 ^d^
	**N (%)**	**LMUP score** ^**a**^	***P*** **value**	
**Apgar 5 min (*n*** = 481) *				
0–3	1	12		
4–6	10 (2.3)	11.5 (11–12)	* 0.135 ^e^	
7–10	470 (97.7)	11 (6–12)		
**Breastfeeding within 1 h (*n*** **= 484)**				
Yes	429 (88.6)	11 (6–12)	0.145 ^b^	
No	55 (11.4)	11 (10–12)		
**Formula feeding (*n*** **= 484)**				
Yes	15 (3.1)	11 (11–12)	*0.152 ^b^	
No	469 (96.9)	11 (6–12)		
**Presence of neonatal complications**				
Yes	121 (25)	11 (6–12)	0.568 ^b^	
No	363 (75)	11 (6–12)		
**Prematurity (*n*** **= 484)**				
Yes	31 (6.4)	11 (7–12)	0.345 ^b^	
No	453 (93.5)	11 (6–12)		
**Jaundice (*n*** **= 484)**				
Yes	22 (4.5)	11 (5.7–12)	0.621 ^b^	
No	462 (95.4)	11 (6–12)		
**Sepsis (*n*** **= 484)**				
Yes	9 (1.8)	10 (4.5–11)	* 0.218 ^b^	
No	475 (98.2)	11 (6–12)		
**Respiratory distress (*n*** **= 484)**				
Yes	36 (7.4)	11 (7.7–12)	0.509 ^b^	
No	448 (92.5)	11 (6–12)		
**Meconium aspiration (*n*** **= 484)**				
Yes	11 (2.3)	10 (3–11)	* 0.16 ^b^	
No	473 (97.7)	11 (6–12)		
**Special care baby unit (SCBU) admission (*n*** **= 484)**				
Yes	70 (14.5)	11 (5–12)	0.816 ^b^	
No	414 (85.5)	11 (6–12)		
**Neonatal Resuscitation(*n*** **= 484)**				
Yes	24 (4.9)	11 (10.3–12)	0.234 ^b^	
No	460 (95.1)	11 (6–12)		

^a^ Data are presented as median [interquartile range]

^b^ Mann–Whitney U tests

^c^ *P* value of Spearman rank correlation test

^d^ Spearman rank correlation between the written characteristics and the LMU*P* value

^e^ Kruskal–Wallis tests

### Psychometric evaluation of Sinhalese version of LMUP

There were 448 study participants who had completed the Sinhalese version of the LMUP and were included for the evaluation of psychometric properties. The psychometric analysis of the Sinhalese LMUP demonstrated excellent internal consistency, with the Cronbach’s alpha score at 0.936. There were very low levels (maximum 2.4%, *n* = 11) of missing values. Item response option endorsement was < 80% and item one has a borderline high endorsement at 80%. All the item-total correlations were above 0.2. Corrected item-total correlations were 0.602 for item 1, 0.907 for item 2, 0.909 for item 3, 0.931 for item 4, 0.931 for item 5, 0.635 for item 6 (Table 7).

**Table 7:** Endorsement and response options for the LMUP scale

Endorsement of the PI items and responses		LMUP in Sinhalese in Sri Lanka				
Items	Category	N	%	Mean	Median	SD
1.At the time of conception	0-always use contraception	24	5.3	1.75	2	0.543
	1-inconsistant /incorrect use contraception	63	14.0			
	2-not use contraception	359	80.1			
	Missing	2	0.4			
2. In terms of becoming a mother	0-wrong time	68	15.1	1.58	2	0.746
	1-ok, but no correct time	45	10.0			
	2-right time	329	73.4			
	Missing	6	1.3			
3. Just before became pregnant	0-not intend to become pregnant	85	18.9	1.54	2	0.802
	1-did not have an idea	29	6.4			
	2-intend to become pregnant	325	72.5			
	Missing	9	2.0			
4. Just before became pregnant	0-not wanted a baby	62	13.8	1.59	2	0.733
	1-have a mixed feeling of baby	49	10.9			
	2-want a baby	326	72.7			
	Missing	11	2.4			
5. Before pregnancy you and your husband	0-never discussed on pregnancy	60	13.3	1.59	2	0.721
	1-discussed on children but no firm agreement	58	12.9			
	2-agreed to pregnancy	322	71.8			
	Missing	8	1.7			
6. Health preparations prior to pregnancy	0-no action	154	34.3	1.02	1	0.848
	1-one action	124	27.6			
	2-two or more action	163	36.3			
	Missing	7	1.0			

The principal component analysis demonstrated that all six items were loaded into one component (Eigenvalues = 4.643) and component loading for item 1 was 0.967, item 2 was 0.946, item 3 was 0.946, item 4 was 0.961, item 5 was 0.959, item 6 was 0.723 (Table 8). We also reported the full range of the LMUP scores (Fig. 1).

**Table 8:** Principal component analysis of LMUP

Items	Missing data	Corrected Item total correlation	Cronbach’s alpha	Cronbach’s alpha if item deleted	Component loadings
Sri Lankan Sinhalese			0.936		Eigenvalues-4.643
1 (Contraception)	2 (0.4%)	.602		.947	.967
2 (Timing)	6 (1.3%)	.907		.912	.946
3 (Desire for motherhood)	9 (2%)	.909		.911	.946
4 (Desire of baby)	11 (2.4%)	.931		.909	.961
5 (Partner discussion)	8 (1.7%)	.931		.909	.959
6 (Preparation)	7 (1.0%)	.635		.950	.723

## Discussion

The proportion of unplanned pregnancy in this study was 17.2% which was lower than the estimated global prevalence of unplanned pregnancy (40%). The proportion of unplanned pregnancies in our study was lower than in South Asian countries such as Nepal (41%), Pakistan (38.2%), Bangladesh (30.3%) [5–7]. The proportion of unplanned pregnancy was higher in our study compared to the developed countries such as the Islamic Republic of Iran (11.1%), Belgium (2%), Kenya (18%), the United Kingdom (16.2%) [16, 17, 51, 56].

Although our results differ from previous Sri Lankan studies which have reported a higher prevalence of unplanned pregnancies, they are consistent with South Asian studies. Community-based study in Bentota was reported 46.7% [45], Colombo was 44% [46] while a hospital-based study in Matara revealed 23.3% [8] unplanned pregnancies. This discrepancy may be due to community-based studies in the early pregnancy period and may have captured more women with unplanned pregnancies. Women with pregnancy loss and pregnancy terminations would have been included in the sample of a community-based study. Pregnancy intentionality may be changed during the pregnancy period, changes in definition and measurement tool of pregnancy intentionality may have contributed to the observed difference.

In this study lower socio-economic status, non- marital relationships, younger age at marriage were significantly associated with a less planned pregnancy. These results were consistent with previous studies in Sri Lanka and other countries. E.g. Malawi, Ethiopia, Pakistan, Bangladesh, Kenya, and Nepal [5–7, 17, 52, 57].

Overall awareness of emergency contraceptives in the current study was 46.4% and unplanned pregnancies were associated with poor awareness of emergency contraceptives (*p* = 0.037). Sri Lankan DHS in 2016 awareness on emergency contraceptives was 53.1% which was consistent with the current research findings [40]. Studies in Pakistan, Bangladesh and, Nepal assessed the overall contraceptive knowledge and it showed that poor knowledge was significantly associated with unplanned pregnancy and reason is self-explanatory [5–7].

This study recorded a strong association between unplanned pregnancy and poor maternity care. Health seeking behavior and prenatal care were suboptimal among women with less planned pregnancies such as delayed booking, fewer than the recommended clinic visits, not attending antenatal classes, peri-conceptional folic acid consumption. Inadequate antenatal care in unplanned pregnancies is also phenomenal in other countries as well [19, 58, 59]. Currently, in the USA, pregnant women with unintended pregnancies were associated with cigarette smoking and lack of adequate vitamins [60]. In Belgium, inadequate folic acid or vitamin intake and a lower number of prenatal clinic visits were significantly associated with an unplanned pregnancy [16].

This finding is not dependent on socio-cultural determinants across the countries as women who not expecting a child would not attend prenatal care to consume folic acid.

Several psychosocial risk factors were associated with an unplanned pregnancy [56]. According to DHS 2016 in Sri Lanka, the prevalence of domestic violence was 17% among ever married women but the current study reported a very low proportion (*n* = 13, 2.6%) and it was a significantly associated factor for less planned pregnancies. Similar results are seen in a study in Auckland, New Zealand, which comprised women identified as victims of physical violence who were more likely to report an unplanned pregnancy than those who were non - victims [61]. This low prevalence of GBV was due to methodological issues, classification of GBV and its sensitive nature. The prevalence of GBV during pregnancy could be lower than the lifetime prevalence of violence.

In the present study, there were 15.5% of pregnant women reported a low mood during the last 2 weeks and it was significantly associated with a less planned pregnancy. The prevalence of antenatal depression was 16.2% in the Anuradhapura district in Sri Lanka [62] and compatible with the current study. A study in Brazil found 25.9% of unplanned pregnant women were suffered from postpartum depression (*p* < 0.05) [63]. In Pennsylvania, the prevalence of postpartum depression was higher among unplanned pregnancies compared to planned pregnancies [64].

This study was limited to the stay period and assesses post partum women one point in time only. Women are stressed and depressed in the immediate post-delivery period due to childbirth experience. The standard timing is to administer EPDS is during the antenatal period and 6 weeks post-partum.

Out of all maternal complications, only maternal anemia (*n* = 39, 19.9%) was associated with a less planned pregnancy. According to DHS in 2016 in Sri Lanka, the prevalence of anemia among pregnant women was 34% [40]. The current study value is far less than the Sri Lankan value and could be due to the over-representation of the Western Province in this study. This association may be due to the confounding effect of the lower socio-economic and educational background associated with an unplanned pregnancy. Poor health knowledge, inadequate clinic participation and inability to obtain iron-rich foods due to financial constraints could have contributed to the anemia experienced by women with an unplanned pregnancy. Previous research suggests that unplanned pregnancies were associated with an increased risk of adverse antenatal, post-natal and birth outcomes [65, 66]. Our research found no significant association with adverse post-partum and neonatal outcomes which was found in the literature [16].

This could be because many Sri Lankan women accept unplanned pregnancy and the health status of women may improve during pregnancy.

Life revolves around the family for most Sri Lankans. Three or four generations often live together in an extended family, with the male side of the family connecting the relations. They could have parents, brothers and sisters with their families and children all living in the same home. Elders are being highly respected by the family far into their old age. In such a living environment, immediate relatives may be more influential to maintain optimal nutrition during pregnancy.

The success history of maternal and newborn programmes with good coverage and community acceptance over the decades were the main contributory factors for newborn indicators in Sri Lanka [39]. Therefore, public health staff was committed to recruiting women with unplanned pregnancies in the service delivery system resulting in more or less optimal newborn health and maternal parameters equivalent to women with a planned pregnancy [67].

These results indicate that the Sinhala LMUP in Sri Lanka performed well, with demonstrated reliability and validity in terms of acceptability, targeting, internal consistency, and structural validity. This validation is comparable to similar validation studies for LMUP conducted in Pakistan, Malawi, India, and Iran [6, 51, 54, 68].

The strength of this study is the use of a psychometrically validated tool to measure the pregnancy planning state. The open and safe atmosphere created for sensitive topics using a self- administered questionnaire of LMUP. Pregnancy planning state assessment was done before delivery, yield more reliable data in the current study. The use of a combination of self-reported interviewed and extracted data from medical records was the strength of this study. A single study is asses several aspects of unplanned pregnancy (proportion, associated factors maternal and newborn health outcomes) resulted in more comprehensive data. This is the first study conducted in Sri Lanka to consider the pregnancy planning state as a continuous variable.

A major limitation of the study was the assessment of pregnancy intentionality on women who are awaiting delivery. Pregnancy intentionality should be ideally ascertained at the time of conception in a community setting to provide more accurate data. The prospective method was not used to conduct the study due to time constraints, although it was ideal. Results may not be generalized to the whole country because of the selection of government sector tertiary care hospital.

Women who were admitted to the gynecologic ward with less than 28 weeks of POA may have been missed in this study. Pregnancy terminations and outcomes in the first two trimesters have been missed in this study. Selection bias could have occurred as sick pregnant women and women with obstetric emergencies were not recruited due to ethical concerns. Capturing of maternal and newborn health outcomes were limited to the duration of hospital stay. Delayed onset complications that could develop after hospital discharge would not be captured in this study.

## Conclusion

Sizeable proportions of pregnancies are unplanned in women delivering at CNTH. Lower socioeconomic status and suboptimal antenatal care were associated with an unplanned pregnancy. According to the current study, the main target group for unplanned pregnancy is socially disadvantaged women. The Sri Lankan public health system needs to focus not only on married women but hard to reach, socially disadvantaged women with high-risk pregnancies. This study highlights the need for strengthening the environment and facilitation for properly planned pregnancies starting from teenagers in Sri Lanka.

## Supplementary information

**Additional file 1.** Questionnaire-English.

**Additional file 2.** Information sheet.

**Additional file 3.** Consent form.

**Additional file 4.** Questionnaire- Sinhalese.

## Data Availability

The datasets that used and analyzed during the current study are available from the corresponding author on reasonable request. As I have no experience in using the public repository and searching a repository for data deposit. I worried about occurring errors, deletions or alterations to data; therefore I will provide data on request.

## Abbreviations

CNTH: Colombo North Teaching Hospital-Ragama
DHS: Demographic and Health Survey
DMPA: Depot medoxy progesterone acetic acid
EPDS: Edinburgh Post-partum depression scale
GBV: Gender-Based Violence
HC: Head Circumference
ICU: Intensive Care Unit
IQR: Interquartile range
IUCD: Intrauterine Contraceptive Device
LMUP: London measure of unplanned pregnancy
OCP: Oral Contraceptive pills
PI: Principle Investigator
PIH: Pregnancy induced hypertension
POA: Period of amenorrhea
PROM: Prolonged rupture of membrane
SCBU: Special Care Baby Unit
SD: Standard deviation
SPSS: Statistical Package for Social Science
USA: United Estate of America
