# Supplementary material for: Proportion of unplanned pregnancies, their determinants and health outcomes of women delivering at a teaching hospital in Sri Lanka
Source: BMC Pregnancy Childbirth. 2020 Nov 5;20:667. doi: 10.1186/s12884-020-03259-2 (PMC7643445; doi:10.1186/s12884-020-03259-2)
Supplement: Supplementary file 1 — Additional file 1. Questionnaire-English. [file 12884_2020_3259_MOESM1_ESM.docx]

**Supplementary file 2**

**PART A:**

**Assessment form for Socio-demographic and obstetric related details**

Interviewer Administered Questionnaire

Ward No: ……………………….

Serial No: ……………………….

Proportion of unplanned pregnancies, their associated factors and health outcomes of women delivering at Colombo North Teaching Hospital -Ragama

Version 01 Dated 29June 2017

**1.-BASIC INFORMATION**

01 What is your BHT no? …………….......

02 what is your LRMP? …………………

03 what is your EDD? ...........................

04 what is your parity? ...........................

**2.-SOCIODEMOGRAGIC INFORMATION**

01 what is your date of birth? …………Year …………..Month ………..Date

02 Age at LRMP in years

03 what is your ethnicity? 04 what is your religion?

.

| 1. Buddhist |
| --- |
| 1. Hindu |
| 1. Islam |
| 1. Catholic |
| 1. Other |

| 1. Sinhala |
| --- |
| 1. Tamil |
| 1. Moor |
| 1. Burger |
| 1. Other |

05 what is your highest educational attainment?

| 1. No schooling | 1. Grade 1-5 |
| --- | --- |
| 1. Grade 6-11 | 1. Passed GCE O/L |
| 1. Grade 12-13 | 1. Passed GCE A/L |
| 1. Tertiary |  |

06 What is your employment?

| 1. No employment | 1. Manager |
| --- | --- |
| 1. Professional | 1. Technician |
| 1. Clerical worker | 1. Service and sales worker |
| 1. Skilled agricultural, Forestry and fishery | 1. Craft and related trades worker |
| 1. Plant, machine operators and assembler | 1. Elementary occupation |
| 1. Armed forces | 1. other(specify) |

07 Childbearing women in Sri Lanka can be married, widowed divorced, cohabiting or unmarried.

What is your marital status?

| 1. Married | 1. Unmarried |
| --- | --- |
| 1. Widowed | 1. Divorced |
| 1. Cohabiting | 1. Single |

08 What is your age at marriage?...................... Years

09 What is your husband’s highest educational attainment?

| 1. No schooling | 1. Grade 1-5 |
| --- | --- |
| 1. Grade 6-11 | 1. Passed GCE O/L |
| 1. Grade 12-13 | 1. Passed GCE A/L |
| 1. Tertiary |  |

10 What is your husband’s employment?

| 1. No employment | 1. Manager |
| --- | --- |
| 1. Professional | 1. Technician |
| 1. Clerical worker | 1. Service and sales worker |
| 1. Skilled agricultural, Forestry and fishery | 1. Craft and related trades worker |
| 1. Plant, machine operators and assembler | 1. Elementary occupation |
| 1. Armed forces | 1. other(specify) |

11 What is your house hold income from all sources? Rs ..............................

12 How do you rate the utilization of family income?

| 1. Difficult to make monthly needs |
| --- |
| 1. Just adequate to make needs |
| 1. Sufficient to make needs |
| 1. More than enough to make needs |

**3.–Health of women and Reproductive Health Assessment**

01 What are the pre-existing medical illnesses you had?

| 1. None | 1. Diabetics mellitus |
| --- | --- |
| 1. Hypertension | 1. Epilepsy |
| 1. Heart disease | 1. Renal disease |
| 1. Asthma | 1. Psychiatric illness |
| 1. other (specify) |  |

If you have any medical illness Go to question C.02

If you don’t have any illness Go to question C.05

02 Did you take any medications for above illness?

Yes No

03 If yes, specify...................................

04 How many living children do you have?

05 what is the age of last child?

06 Have you ever use a family Planning method?

Yes go to question 15

No go to question 24

07 If yes, what was the most recent family planning method you used prior to this pregnancy?

| 1. Natural methods | 1. Pills |
| --- | --- |
| 1. Condoms | 1. DMPA |
| 1. IUCD | 1. Implants |
| 1. Other |  |

C.08 why did you use most recent family planning method?

| 1. as we completed the family |
| --- |
| 1. to space the pregnancy |
| 1. as we didn’t want children |
| 1. other |

09 Whom did you get advice on how to use that method?

Doctor Health care worker friend Media

10 Were you ever told by a health worker about other methods of family planning that you could use?

Yes No

11 Did you discontinue the most recent method you used?

Yes No

12 If yes what is the reason? (Number most appropriate reasons, could be more than one answer)

| 1. Infrequent sex | 1. Became pregnant while using |
| --- | --- |
| 1. Wanted to became pregnant | 1. Husband /partner disapproved |
| 1. Waned more effective method | 1. Health concerns |
| 1. Due to side effects | 1. Lack of access /too far |
| 1. Cost is too much | 1. inconvenient |
| 1. Marital dissolution | 1. Other |

Please go to the question C 14

13 If you did not use family planning method, why you didn’t use? (Number most appropriate reasons, could be more than one answer)

| 1. Wanted to become pregnant | 1. Lack of knowledge or lack of sources |
| --- | --- |
| 1. I don’t like to FP | 1. Husband disapproves |
| 1. Other people disapproves | 1. Infrequent sex |
| 1. Health concerns | 1. Problem of access/availability |
| 1. Cost too much | 1. Religion reasons |
| 1. inconvenient to use | 1. Rumours of side effects |
| 1. Other | 1. Don’t know |

| Yes |  |
| --- | --- |
| No |  |

14 Have you ever heard of emergency contraceptive methods (postinor)?

15 If yes did you ever use it?

| Yes |  |
| --- | --- |
| No |  |

**4. –ANTENATAL CHARACTERISTICS**

01 When did you confirm your pregnancy?

02 What was your POA at booking visit?

03 When did you start folic acid supplementation?

1. Preconception
2. At the time I confirm pregnancy
3. After the confirmation of pregnancy

05 How did you rate your compliance to nutritional supplements?

a. Poor b. Fair c. Average d. Good e. Excellent

| 06 How many antenatal clinics did you attend? |  | |  | |
| --- | --- | --- | --- | --- |
| 07 How many antenatal classes did you attend? |  | |  | |
| 08 Did you maintain kick count chart | Yes |  | No |  |

09 What were the medical illnesses you had during this pregnancy?

| 1. None | 1. Hyperemesis gravidarum |
| --- | --- |
| 1. Gestational diabetics | 1. Bleeding (antepartum) |
| 1. Pregnancy induced hypertension | 1. Urinary tract infection |
| 1. Anemia | 1. Preterm contractions |
| 1. Premature Rupture of membrane | 10. other |

**5.–Psychosocial Risk Factors**

1. Did you suffer any mental health problem prior to pregnancy? Yes No

1. Have you ever tried to do self-harm? Yes No

1. Have you ever take psychoactive substance / smoked? Yes No
2. Does your husband ever smoke during last year? Yes No
3. Do your husband usually take /psychoactive substance/alcohol? Yes No
4. If yes, are you having difficulties in your life as a result of his alcohol intake? Yes No

1. Since you were pregnant, have you been abused by husband? Yes No
2. Since you have been pregnant what are the types of violence you had by husband?

Emotional Physical

| 9. How do you rate the relationship you had with your husband?  a. Poor b. Fair c. Average d. Good e. Excellent |
| --- |
|  |
| 10. How do you rate the relationship you had with family members?  a. Poor b. Fair c. Average d. Good e. Excellent |

1. Do you have any physically or psychologically handicapped babies? Yes No
2. . Did you have low mood for the last two weeks?

Yes No

1. Did you having lack of interest in usual pleasurable activities?

Yes No

1. Were you unable to carry out daily functions?

Yes No

1. Did you experience of any stressful life events in the last 6 months ?( death, unemployment, separation)

Yes No

**PART B- ASSESSMENT OF PLANNING SATATUS CURRENT PREGNANCY**

Below are some questions that ask about your circumstances and feelings around the time you became pregnant. Please think of your most recent pregnancy when answering the questions below. For every question, there are possible responses. Please read all the responses and then choose the one option that is applicable to you and your husband.

1. In the month that I became pregnant...... I/we were not using contraception

I/we were using contraception, but not on every occasion

I/we always used contraception, but knew that the method had failed (i.e. broke, moved, came out, not worked) at least once

I/we always used contraception

1. In terms of becoming a mother (first time or again),I feel that my pregnancy happened at the......

Right time

Ok, but not quite right time

Wrong time

1. Just before I became pregnant.......

I intended to get pregnant

My Intention kept changing

I did not intend to get pregnant

1. Just before I became pregnant.......

I wanted to have a baby

I had mixed feelings about having a baby

I did not want to have a baby

5. Before I became pregnant... (please tick the statement which most applies to you)

My partner and I had agreed that we would like me to be pregnant

My partner and I had discussed having children together, but hadn’t agreed for me to get pregnant we never discussed having children together

6. Before you became pregnant, did you do anything to improve your health in preparation for pregnancy? (Please tick all that apply)

Took folic acid Stopped or cut down smoking

Stopped or cut down drinking alcohol Ate more healthily

Sought medical/health advice took some other action, please describe

I did not do any of the above before my pregnancy

**PART C : Maternal and Newborn data extraction sheet**

Ward No: ……………………….

Serial No: ……………………….

Data Extraction Form

BHT No.........................................

Proportion of unplanned pregnancies, their associated factors and health outcomes of women delivering at Colombo North Teaching Hospital–Ragama

**1. - INTRAPARTUM AND POSTPARTUM OUTCOMES**

01 Best estimate of POA at delivery? ...................weeks …….days

02 What was the onset of labor?

1. Spontaneous

2. Induced

03 Were there Features of fetal distress?

Yes No

1. Meconium stained liquor
2. CTG deceleration/nonreactive

04 What was the duration of labor?

1. <6 hours 2. 6-12hrs

3. 12-18hrs 4. >18hrs

05 what was the mode of delivery?

1. Vaginal 2. Instrumental

3. ELLSCS 4. EMLSCS

06 What were the maternal intrapartum /postpartum complications?

| 1. None |  | 1. Post-partum Hemorrhages |  |
| --- | --- | --- | --- |
| 1. Perineal laceration |  | 1. Fever >100F |  |
| 1. Elevated blood pressure |  | 1. Wound dehiscence |  |
| 1. Post-operative wound infection |  | 1. ICU admission |  |
| 1. Fits |  | 1. Difficulty in passing urine |  |
| 1. Shoulder dystocia |  | 1. Other(Specify) |  |

07 How many days did you stay in the Hospital?

**2. NEONATAL OUTCOMES**

01 What is the condition of the baby?

| 1. Live birth |
| --- |
| 1. Stillbirth>24wks |
| 1. Intrauterine death<24wks |
| 1. Intrapartum death |
| 1. Neonatal death |

02 What was the Apgar score of baby? 1min 5min 10min

| 03 What is the sex of baby ? | Male | female |
| --- | --- | --- |
| 04 What is the birth weight of the baby? |  | grams |
| 05 What is the Head circumference of the baby? |  | cm |
| 06 Did you breastfeed the baby within one hour of birth? | yes | No |

07 If no why?

| 1. Mother had complications |  | 1. Baby had complications |  |
| --- | --- | --- | --- |
| 1. Poor sucking |  | 1. Inadequate breast milk |  |

| 08 Did you give formula milk to the baby? | Yes | No |
| --- | --- | --- |
| 09 Was the baby resuscitated after birth? | Yes | No |

10 What were the neonatal outcomes?

| 1. None |  | 1. Prematurity<37 weeks |  |
| --- | --- | --- | --- |
| 1. Neonatal jaundice |  | 1. Sepsis/meningitis |  |
| 1. Respiratory difficulties |  | 1. Congenital heart disease |  |
| 1. Fever>100F |  | 1. meconium aspiration |  |
| 1. Macrosomia>3.5 kg |  | 1. Intrauterine growth restriction |  |
| 1. Birth Asphyxia |  | 1. Congenital anomaly |  |
| 1. PBU admission |  | 1. other |  |

London Measure of Unplanned Pregnancy –Scoring guidelines

| Introduction | Now I am going to ask you a few questions about your circumstances and feelings around the time you became pregnant. Please think of your current pregnancy when answering these questions. For every question, there are possible responses to it. Please read all the responses and then choose the one option that is most applicable to you. |
| --- | --- |
| Item 1 (contraception) | In the month that you became pregnant, you and your husband… READ RESPONSES 1-4 ONLY (MARK ONLY ONE RESPONSE) …were not using a method of pregnancy prevention (2 points) …were using a method of pregnancy prevention, but not on every occasion (1 point) …always used a method of pregnancy prevention, but this method did not work on some occasions (i.e. broke, moved, came off) (1 point) …always used a method of pregnancy prevention (0 points) |
| Item 2 (timing) | Now I am going to ask you a question and there are 4 possible responses to it. Please choose the one option that is applicable to you . Do you feel that this pregnancy happened at the time you … READ RESPONSES 1-4 ONLY (MARK ONLY ONE RESPONSE) …wanted pregnancy then (2 points) …wanted pregnancy sooner (2 points) …wanted pregnancy later (1 point) …did not want to be pregnant at all (0 points) |
| Item 3 (intention) | Now I am going to ask you a question and there are 3 possible responses to it. Please choose the one option that is applicable to you.. Think about the time just before you became pregnant. Would you say… READ RESPONSES 1-3 ONLY (MARK ONLY ONE RESPONSE)  …you intended to get pregnant (2 points) …you did not intend to get pregnant (0 points) …your intentions to get pregnant kept changing (1 points) |
| Item 4 (want) | Now I am going to ask you a question and there are 3 possible responses to it. Please choose the one option that is applicable to you. Again think about the time just before you became pregnant and say… READ RESPONSES 1-3 ONLY (MARK ONLY ONE RESPONSE) …you wanted to have a baby (2 points) …you had mixed feelings about having a baby (1 points) …you did not want to have a baby (0 points) |
| Item 5 (discuss) | Now I am going to ask you a question and there are 3 possible responses to it. Please choose the one option that is applicable to you. Now think about you and your husband before you became pregnant. READ RESPONSES 1-3 ONLY (MARK ONLY ONE RESPONSE) …you and your husband had agreed that you would like for you to be pregnant (2 points) …you and your husband had discussed having children together, but hadn’t agreed for you to get pregnant (1 points) …you and your husband had never discussed having children together (0 points) |
| Item 6 (prepare) | Before you became pregnant, you did the following things in preparation for pregnancy: READ ALL RESPONSES AND CHECK ALL THAT APPLY …took vitamins …stopped or cut down on smoking, drinking, or eating paan leaves …changed your diet, such as ate more healthfully or avoided hot foods …sought medical or health advice or information about pregnancy …saved money for medical expenses …took some other action: ______________ (Add checked responses. Score is 0 checks=0 points, 1 check=1 point, ≥2 checks=2 points.) |
