# Supplementary material for: Proportion of unplanned pregnancies, their determinants and health outcomes of women delivering at a teaching hospital in Sri Lanka
Source: BMC Pregnancy Childbirth. 2020 Nov 5;20:667. doi: 10.1186/s12884-020-03259-2 (PMC7643445; doi:10.1186/s12884-020-03259-2)
Supplement: Supplementary file 2 — Additional file 2. Information sheet. [file 12884_2020_3259_MOESM2_ESM.docx]

**PATIENT INFORMATION SHEET**

**Proportion of unplanned pregnancies, their associated factors and health outcomes of women delivering at Colombo North Teaching Hospital -Ragama**

I am Dr…………………………………… attached to the post graduate institute of Medicine University of Colombo. My current designation is PGIM trainee in MSc Community medicine. I would like to invite you to take part in the research project titled “Proportion of unplanned pregnancies, their associated factors and health outcomes of women delivering at Colombo North Teching Hospital -Ragama”Conducted by Dr ………………………………….at Colombo North Teaching Hospital -Ragama.

**1. Purpose of the study**

This research project aim to determine the proportion, associated factors of unplanned pregnancies which ending at birth and impact of unplanned pregnancy on maternal and newborn health. This information will assist in identify burden of unplanned pregnancies and to design programs for prevent such pregnancies. Health consequences of unplanned pregnancies will be compared with planned pregnancies. This is the first study in Sri Lanka regarding this matter.

**2. Voluntary participation**

Your participation in this study is voluntary. You are free not to participate at all or to withdraw from the study at any time despite consenting to take part earlier. There will be no loss of medical care for you and baby if you entitle to this research. If you decide not to participate or withdraw from the study you may do so at any time.

**3. Participant Selection**

Study participants will be postnatal mothers who are fit to be discharged according to inclusion and exclusion criteria. Details of pregnancy planning state as well as both maternal and fetal health outcomes can be obtained easily in postnatal setting. Post-partum mothers will be selected according to Birth Registry where both live and non-live deliveries documented.

**4. Duration, procedures of the study and participant’s responsibilities**

Once you consented, data will be collected by a medical officer it will take 30 minits. Investigator will administer a self-administered questionnaire and then interviewer administered questionnaire. Some relevant details obtain from pregnancy record and Bead Head tickets and neonatal examination formats.

**5. Potential benefits**

Participation in this study may improve their knowledge on reproductive health. Results of this research will be published in journals and presented at conferences will helpful to implement strategies to minimize the unplanned pregnancies and early identification specialized care provision.

**6. Risks, hazards and discomforts**

Participation in this study will not entitle to any fine, withholding of health and only you have to spend 30 minutes from your valuable time.

**7. Confidentiality**

Confidentiality of all records is guaranteed and no information by which you can be identified will be released and only anonymous data will be published. You will not be identifiable in any reports, publications arising from this research. You are free to ask any question at any time from the investigator.

**8. Sharing the Results**

Only scientific outcome of this study will be shared with journals, presentations and scientific publications. No confidential details will be shared for any reason.

**9. Termination of study participation**

You may withdraw your consent to participate in this study at any time, with no penalty or effect on medical care or loss of benefits. Please notify the investigator as soon as you decide to withdraw your consent.

**10. Clarification**

If you need any clarification and further information please feel free to contact me at any time.my contact details are given below.

Investigator

Dr…………………………………………….

Post Graduate Trainee

Post Graduate Institute of medicine

160, Prof .Nandadasa Kodagoda Mawatha,

Colombo 7

Telephone-0112696261/0766915152

Thank you for your interest in the study.

**………………………………………..**
