# Supplementary material for: Proportion of unplanned pregnancies, their determinants and health outcomes of women delivering at a teaching hospital in Sri Lanka
Source: BMC Pregnancy Childbirth. 2020 Nov 5;20:667. doi: 10.1186/s12884-020-03259-2 (PMC7643445; doi:10.1186/s12884-020-03259-2)
Supplement: Supplementary file 3 — Additional file 3. Consent form. [file 12884_2020_3259_MOESM3_ESM.docx]

**CONSENT FORM**

**Proportion of unplanned pregnancies, their associated factors and health outcomes of women delivering at Colombo North Teaching Hospital -Ragama**

**To be completed by the participant**

The participant should complete the whole of this sheet himself/herself.

| 1. Have you read the information sheet? (Please keep a copy for yourself) | YES/NO |
| --- | --- |
| 2. Have you had an opportunity to discuss this study and ask any questions? | YES/NO |
| 3. Have you had satisfactory answers to all your questions? | YES/NO |
| 4. Have you received enough information about the study? | YES/NO |
| 5. Who explained the study to you? | …………………… |
| 6. Do you understand that you are free to withdraw from the study at any time, without having to give a reason and without affecting your future medical care? | YES/NO |
| 7. Medical records and other data by Investigators relating to your participation in this study may be examined by other research assistants. All personal details will be treated as **strictly Confidential**. Do you give your permission for these individuals to have access to your records? | YES/NO |
| 8. Have you had sufficient time to come to your decision? | YES/NO |
| 9. Do you agree to take part in this study? | YES/NO |

Participant’s signature…………………………..………… Date…………………….

Name (BLOCK CAPITALS)…………………………………………………………

**To be completed by the investigator/ person obtaining consent**

I have explained the study to the above volunteer and he/ she has indicated her willingness to take part.

Signature of investigator……………………....………….. Date……………………….

Name (BLOCK CAPITALS)……………………………………………………….
