# Supplementary material for: Proportion of unplanned pregnancies, their determinants and health outcomes of women delivering at a teaching hospital in Sri Lanka
Source: BMC Pregnancy Childbirth. 2020 Nov 5;20:667. doi: 10.1186/s12884-020-03259-2 (PMC7643445; doi:10.1186/s12884-020-03259-2)
Supplement: Supplementary file 4 — Additional file 4. Questionnaire- Sinhalese. [file 12884_2020_3259_MOESM4_ESM.pdf]

වාට්ටු අංකය :
අනු අංකය :
ප්‍රශ්නාවලිය ඉදිරිපත් කරන්නාගේ නම :

උතුරු කොළඹ ශික්ෂණ රෝහලේ සිදුවන පෙර - සැලසුම් නොකර සිදු වූ ගර්භනීභාවයන් ගණනය කිරීම, එයට අදාල කරුණු සහ දරු ප්‍රසූතියෙන් පසු ඇතිවන ප්‍රතිඵල සොයා බැලීමේ අධ්‍යයනය

පළමු සංස්කරණය 2017 ජුනි 29.

1 කොටස

අ - මූලික තොරතුරු

1. ඔබට අන්තිමට ක්‍රමවත්ව ඔසප් වූ දිනය? ☐
2. දරුවා උපදින විට ගර්භයට සති ගණන? ☐
3. මේ ඔබගේ කී වෙනි ගර්භයද? ☐

ආ - සාමාජීය තොරතුරු

01. ඔබගේ සෞඛ්‍ය වෛද්‍ය නිලධාරී කොට්ඨාශය කුමක්ද? ☐
02. ඔබගේ පසුගිය උපන් දිනයට වයස අවුරුදු වලින් කොපමණද? ☐
03. ඔබට අවසානයට ඔසප් වූ දිනයට වයස? ☐
04. ඔබගේ ජාතිය කුමක්ද?
   1) සිංහල ☐
   2) දෙමළ ☐
   3) මුස්ලිම් ☐
   4) බර්ගර් ☐
   5) වෙනත් ☐
05. ඔබගේ ආගම කුමක්ද?
   1) බෞද්ධ ☐
   2)හින්දු ☐
   3) ඉස්ලාම් ☐
   4) කතෝලික ☐
   5)වෙනත් ☐

06. ඔබ ලැබූ උසස්ම අධ්‍යාපන මට්ටම කුමක්ද?
   1) පාසල් අධ්‍යාපනය ලබා නැත. ☐ 2)ප්‍රාථමික පාසල් අධ්‍යාපන ලබා ඇත. ☐
   3)6-1 වසර දක්වා ☐ 4)අ.පො.ස. (සා/පෙළ) සමත්. ☐
   5)අ.පො.ස. (උ/පෙළ) දක්වා. ☐ 6)අ.පො.ස. (උ/පෙළ) සමත්. ☐
   7)ඩිප්ලොමා / උපාධි සමත්. ☐

07. ඔබගේ රැකියාව කුමක් ද?
   1)විධායක / වෘත්තිය ☐ 2)ගුරු / හෙද / ලිපිකරු ☐
   3)ආරක්ෂක අංශ ☐ 4)වෙළඳ ව්‍යාපාරික ☐
   5)කෘෂිකාර්මික ☐ 6)පුහුණු කම්කරු ☐
   7)නුපුහුණු කම්කරු ☐ 8)රැකියාවක් නොමැත ☐

ශ්‍රී ලංකාවේ දරුවන් උපදින කාන්තාවන් විවාහක, අවිවාහක, දික්කසාද, එකට ජීවත් වන්නන් විය හැක.

08. ඔබ මින් කුමන ගනයට අයත් වන්නේ ද?
   1)විවාහක ☐ 2)අවිවාහක ☐ 3)වැන්දඹු ☐
   4)දික්කසාද ☐ 5)එකට ජීවත් වේ ☐ 6)තනිව ජීවත් වේ ☐

09. ඔබ විවාහ වන විට වයස අවුරුදු කීයද? ☐

ඔබ විවාහක නම් ඔබගේ ස්වාමි පුරුෂයාගේ තොරතුරු පහත සඳහන් කරන්න.

10. ස්වාමි පුරුෂයාගේ අධ්‍යාපන මට්ටම කුමක්ද?

1) පාසල් අධ්‍යාපනය ලබා නැත. ☐ 2)ප්‍රාථමික පාසල් අධ්‍යාපන ලබා ඇත. ☐
3)6-1 වසර දක්වා ☐ 4)අ.පො.ස. (සා/පෙළ) සමත්. ☐
5)අ.පො.ස. (උ/පෙළ) දක්වා. ☐ 6)අ.පො.ස. (උ/පෙළ) සමත්. ☐
7)ඩිප්ලොමා / උපාධි සමත්. ☐

11. ඔබගේ ස්වාමි පුරුෂයාගේ රැකියාව කුමක්ද?

1)විධායක / වෘත්තිය ☐ 2)ගුරු / හෙද / ලිපිකරු ☐
3)ආරක්ෂක අංශ ☐ 4)වෙළඳ ව්‍යාපාරික ☐
5)කෘෂිකාර්මික ☐ 6)පුහුණු කම්කරු ☐
7)නුපුහුණු කම්කරු 8)රැකියාවක් නොමැත ☐

12. ඔබගේ පවුලේ මාසික ආදායම දල වශයෙන් රුපියල් කීයද?

1) <10,000 ☐ 2) 10,000 - 20,000 ☐ 3) 20,000 - 30,000 ☐
4) 30,000 - 40,000 ☐ 5) >40,000 ☐

13. ඔබගේ පවුලේ ආර්ථික තත්වය කෙසේද?

1) මාසික අවශ්‍යතා සපුරාලිමට ප්‍රමාණවත් නොවේ. ☐
2) මාසික අවශ්‍යතා සපුරාලීමට යාන්තම් ප්‍රමාණවත් වේ. ☐
3) මාසික අවශ්‍යතා සපුරාලීමට ප්‍රමාණවත් වේ. ☐
4) මාසික අවශ්‍යතා සපුරාලීමට හොදාකාරව ප්‍රමාණවත් වේ. ☐

14. ඔබ ගර්භනී වීමට පෙර ස්ථාවර පදිංචි ස්ථානයක් තිබුනේද? ඔව් ☐ නැත ☐
☐ ☐

ඇ - ඔබගේ සෞඛ්‍ය හා ගර්භනීභාවයේ අවධානම් තත්වය

01. ඔබ ගර්භනී වීමට පෙර සිට පැවතී රෝගී තත්වයන් මොනවාද?

1) දියවැඩියාව ☐ 2) අධි රුධිර පීඩනය ☐ 3) උපතේ සිට ඇති හෘද රෝග ☐
4) හෘදයාබාද ☐ 5) ඇදුම ☐ 6) අපස්මාරය ☐
7) වකුගඩු රෝග ☐ 8) මානසික රෝග ☐ 9) වෙනත් ☐

02. ඔව් නම් ඒ සඳහා ප්‍රතිකාර ලබා ගත්තාද? ඔව් ☐ නැත ☐

03. ඔව් නම් ඒ මොනවාද? ....

04. ඔබ එම ප්‍රතිකාර ලබාගත් ආකාරය?

1. වඩාත් අක්‍රමවත් ☐ 2. අක්‍රමවත් ☐ 3. සමාන්‍යයි. ☐ 4. ක්‍රමවත් ☐ 5. වඩාත් ක්‍රමවත් ☐

05. ඔබට කුමන ආකාරයේ හෝ මානසික අසනීප තත්වයකින් පෙළුනේද? ඔව් ☐ නැත ☐

06. ඔබ ඔබට අනතුරක් කර ගැනීමට උත්සහ කර තිබේද? ඔව් ☐ නැත ☐

07. ඔව් නම්,

මෙම ගර්භනීභාවයට පෙර ☐ මෙම ගර්භනී අවධියේ ☐

08. ඔබගේ ස්වාමි පුරුෂයා පසුගිය අවුරුද්ද ඇතුලත දුම් පානය කලේද? ඔව් ☐ නැත ☐

09. ඔබ දුම් පානය කලේද? ඔව් ☐ නැත ☐

10. ඔව් නම්,

මෙම ගර්භනීභාවයට පෙර ☐ මෙම ගර්භනී අවධියේ ☐

11. ඔබගේ ස්වාමි පුරුෂයා මධ්‍යසාර පාවිච්චි කරන්නේද? ඔව් ☐ නැත ☐

12. ඔව් නම් ඒ ඔබගේ ජීවිතයට කරදරකාරී මට්ටමකින්ද? ඔව් ☐ නැත ☐

13. ඔබ මධ්‍යසාර පාවිච්චි කරන්නේද? ඔව් ☐ නැත ☐

14. ඔව් නම්,

මෙම ගර්භනීභාවයට පෙර ☐ මෙම ගර්භනී අවධියේ ☐

15. ඔබට පසුගිය සති දෙක ඇතුළත අසතුටින් පසුවුවේද? ඔව් ☐ නැත ☐

16. ඔබට සතුටුදායක අවස්ථාවලට ආශාවක් තිබුනේ නැද්ද? ඔව් ☐ නැත ☐

17. ඔබට කිසිවෙකු ලඟ නැතිවිටදී ශබ්ද ඇසුනේද? ඔව් ☐ නැත ☐

18. ඔබට අතීතයේදී හෝ වර්තමානයේදී අසාමාන්‍ය අත්දැකීමකට මුහුණ දුන්නේද? ඔව් ☐ නැත ☐

19. ඔබට ඔබගේ එදිනෙදා කටයුතු කර ගැනීමට අපහසුද? ඔව් ☐ නැත ☐

20. ඔබට ඔබගේ දරුවාගේ කටයුතු කර ගැනීමට අපහසුද? ඔව් ☐ නැත ☐

21. ඔබට පසුගිය මාස 6 ඇතුළත කුමන හෝ ආතතියක් ගෙන දෙන අත්දැකීමටකට මුහුණු දුන්නේද?
(මරණය, රැකියාව අහිමි වීම, වෙන් වීම) ඔව් ☐ නැත ☐

22. ඔබට කායික හෝ මානසික රෝගී තත්වයක් ඇති දරුවන් සිටීද? ඔව් ☐ නැත ☐

23. ඔබ ඔබගේ ස්වාමී පුරුෂයා සමඟ ඇති සම්බන්දතාවය අර්ථ දක්වන්නේ කෙසේද?

දුර්වල ☐ තරමක් දුරට ☐ සමාන්‍යයි ☐ හොඳයි ☐ ඉතා හොඳයි ☐

24. ඔබ ඔබගේ පවුලේ සමාජිකයන් සමඟ ඇති සම්බන්දතාවය අර්ථ දක්වන්නේ කෙසේද?

දුර්වල ☐ තරමක් දුරට ☐ සමාන්‍යයි ☐ හොඳයි ☐ ඉතා හොඳයි ☐

25. ගර්භනීභාවයට පෙර ඔබ ඔබගේ ස්වාමී පුරුෂායාගේ වධ හිංසා වලට ලක් වුවේද? ඔව් ☐ නැත ☐ ☐

26. ඔව් නම් ඒ කුමන ආකාරයෙන්ද? මානසික ☐ කායික ☐

27. ගර්භනී වූ පසු ඔබ ඔබගේ ස්වාමී පුරුෂයාගේ වධ හිංසා වලට ලක් වුවේද? ඔව් ☐ නැත ☐

28. ඔව් නම් ඒ කුමන ආකාරයෙන්ද? මානසික ☐ කායික ☐

29. ඔබ පසුගිය වසර තුල කිසිවෙකු ඔබට තර්ජනාත්මකව ලිංගික අතරවර සිදු කලේද? ඔව් ☐ නැත ☐

ඈ - ඔබගේ පසුගිය ගර්භනී භාවයන් පිළිබඳ තොරතුරු

01. ඔබ විවාහයෙන් හෝ අවසාන ජීවත්ව සිටින දරුවාගෙන් පසු දරුවන් කොපමණ අපේක්ෂා කලේද? ☐

02. ඔව් නම් විවාහයෙන් හෝ අවසාන ජීවත්ව සිටින දරුවාගෙන් පසු කොපමණ කාලයට පසුවද? අවු ☐ මාස ☐

03. ඔබගේ එක් එක් ගර්භනී භාවයන් පහත ආකාරයට විස්තර කරන්න.

	සජීවී උපන් / අජීවී උපන්	පෙර සැලසුම් කර ඇත / නැත	ස්ත්‍රී / පුරුෂ භාවය	උපන් ආකාරය	උපත් බර	ගර්භයට සති ගණන	සංකූලතා
පළමු ගර්භය							
දෙවන ගර්භය							
තෙවන ගර්භය							
හතරවන ගර්භය							
පස්වන ගර්භය							

04. මෙම දරුවා පිළිසිඳ ගැනීම සිදුවන විට ඔබ කුමන හෝ උපත් පාලන ක්‍රමයක් භාවිත කලේද?
ඔව් ☐ නැත ☐

05. ඔව් නම් එම උපත් පාලන ක්‍රමය කුමක්ද?
1) ස්වාභාවික ☐ 2) පෙති ☐ 3) කොන්ඩම් ☐
4) එන්නත් ☐ 5) ලූපයක් දැමීම ☐ 6) ජඩෙල් ස්ථාපිත කිරීම ☐

06. නැත නම් ඒ කුමන හේතු මතද?
1) දරුවකු බලාපොරොත්තු විය. ☐ 2) ඒ සඳහා වේලාවක් තබුනේ නැත. ☐
3) ඒ සම්බන්දව සිතා සිටියේ නැත. ☐ 4) අපට දරුවන් ලැබේ යැයි සිතා සිටියේ නැත. ☐
5) ඒ පිළිබඳව දැන සිටියේ නැත. ☐ 6) අතුරු ආබාද නිසා පාවිච්චි කලේ නැත. ☐
7) විශ්වාස නිසා භාවිතා කිරීමට භය විය ☐ 8) වෙනත් ☐

දැනට ශ්‍රී ලංකාවේ ගබ්සා කිරීම් විශාල වශයෙන් ව්‍යාප්ත වී ඇත.

07. ඔබත් එවැනි ගබ්සා කිරීම් ගැන කෙදිනක හෝ සිතා සිටියේද? ඔව් ☐ නැත ☐

08. ඔබත් එවැනි ගබ්සා කිරීමක් කෙදිනක හෝ කිරීමට උත්සාහ කර තිබේද? ඔව් ☐ නැත ☐

ඉ කොටස - මෙම ගර්භනී භාවය පිළිබඳ තොරතුරු

01. ඔබ ගර්භනී බව දැනගන්නා විට ගර්භයට සති කියද? ☐

02. ඔබ පළමු මාතෘ සායනයට පැමිණෙන විට ගර්භයට සති කීයද? ☐

03. මුල් මාතෘ සායනයේ දී ඔබගේ ශරීර ස්කන්ධ දර්ශකය කීයද? ☐

04. ගර්භනී සමයේදී ඔබගේ බර වැඩිවීමට කොපමණද?

05. ඔබ ගර්භනී සමයේදී පහත ඖෂධ ලබා නොගත්තේද?

1) ෆොලික් අම්ලය ☐ 2) යකඩ ☐ 3) කැල්සියම් ☐

06. නැත්නම් ඒ කුමන හේතු නිසාද?

1) සැලකිලිමත් වු නැති නිසා ☐ 2) මාගේ සෞඛ්‍ය හොද නිසා ☐ 3) දැන සිටියේ නැත ☐

4) ශරීරයට අපහසු නිසා ☐ 5) කිසිවෙකු නොකී නිසා ☐ 6) වෙනත් ☐

07. ඔබ ප්‍රදේශයේ මාතෘ සායනයට සහභාගී වූ දින ගණන කීයද? ☐

09. ඔබ කොපමණ පූර්ව, පසුව සැසි සඳහා සහභාගී වූයේ ද? ☐

09. ඔබ දරුවා දගලන වාර ගණන සටහන් කලේද? ඔව් ☐ නැත ☐

10. ඔබ දරු ප්‍රසූතිය සඳහා පෙර සිට සූදානම් වූයේ කෙසේද?

1) රෝහල තෝරාගෙන තිබුනි ☐ 2) ප්‍රවාහන ක්‍රමයක් තිබුනි ☐ 3) වියදම තිරණය කර තිබුනි ☐

4) රෝහලට ඇති දුර හා ලඟ වීමට ගත වනකාලය දැන සිටියෙමි ☐

11. ගර්භනී සමයේදී පහත සඳහන් රෝගී තත්ව ඔබට තිබුනේද?

1) අධික වමනය ☐ 2) දියවැඩියාව ☐

3) ප්‍රසූතියට පෙර පටුල බිදීම ☐ 4) ප්‍රසූතියට පෙර ගර්භයේ සංකෝචන ☐

5) අධි රුධිර පීඩනය / මුත්‍රා සමඟ ප්‍රෝටීන් පිටවීම ☐ 6) මුත්‍රා ආසාධානයන් ☐

7) රක්ත හීනතාවය ☐ 8) වෙනත් ☐

වාට්ටු අංකය :

අනු අංකය :

දත්ත උපුටා ගැනීමේ පත්‍රිකාව

උතුරු කොළඹ ශික්ෂණ රෝහලේ සිදුවන පෙර - සැලසුම් නොකර සිදු වු ගර්භනීභාවයන් ගණනය කිරීම, එයට අදාල කරුණු සහ දරු ප්‍රසුතියෙන් පසු ඇතිවන ප්‍රතිඵල සොයා බැලිමේ අධ්‍යයනය

පළමු සංස්කරණය 2017 ජුනි 29.

2 කොටස

අ- දරු ප්‍රසුතියේ හා ඉන් පසු තොරතුරු

01. උපත ආරම්භ වූයේ කෙසේද? ස්වභාවිකව ☐ කෘතීමව ☐

02. දරු ප්‍රසුතිය සඳහා කොපමණ වෙලාවක් ගත වූයේද?
    1) පැය 6 ට අඩු ☐ 2) පැය 6 - 12 ☐ 3) පැය 12 - 18 ☐ 4) පැය 18 ට වැඩි ☐

03. දරු උපත සිදු වූයේ කුමන ආකාරයටද?
    1) ස්වභාවික මාර්ගයෙන් ☐ 2) උපකරණ භාවිතයෙන් ☐
    3) සිසේරියන් සැත්කමින් ☐ 4) හදිසි සිසේරියන් සැත්කමින් ☐

04. ගර්භයේ තිබූ දියරයේ ස්වාභාවය කෙසේද?
    1) පැහැදිලි ☐ 2) මිකෝනියම් ☐ 3) ආසාදිතය ☐

05. ප්‍රසුතියේදී හා ප්‍රසුතියෙන් පසු ඔබ හට තිබූ සංකූලතා මොනවාද?
    1) උණ ≥ 100 ☐ 2) රුධිර වහනය ☐
    3) යෝනි මාර්ගයේ තුවාල වීම ☐ 4) දරුවා සිර වීම ☐
    5) අධි රුධිර පීඩනය ☐ 6) තුවාල වෙන්වීම ☐
    7) තුවාල ආසාධනය ☐ 8) දැඩි සත්කාර ඒකකයට ඇතුල් වීම ☐
    9) අපස්මාර ☐ 10) මුත්‍රා පිටවීමේ ප්‍රශ්න ☐
    11) වෙනත් ☐

06. ඔබ කොපමණ දින ගණනක් වාට්ටුවේ නතර වූයේද? ☐

ආ - ළදරුවා සම්බන්ධ තොරතුරු

01. ඉපදෙන විට දරුවාගේ තත්වය කුමක්ද?

1) සජීවී උපත් ☐ 2) සති 24ට අඩු මල දරු උපත් ☐
3) සති 24ට වැඩි මල දරු උපත් ☐ 4) උපතේදී මිය යාම ☐
5) උපතෙන් පසුව මිය යාම ☐

02. ඔබගේ දරුවා ගැහැණු දරුවෙක්ද? පිරිමි දරුවෙක්ද? ☐

03. දරුවාගේ ඇප්ගා අගය කුමක්ද? 1) 1 විනාඩි ☐ 2) 5 විනාඩි ☐ 3) 10 විනාඩි ☐

04. දරුවාගේ උපත් බර කොපමණද? ☐

05. දරුවාගේ හිසේ වට ප්‍රමාණය කොපමණද? ☐

06. ඉපදී පැයක් ඇතුලත දරුවාට මව් කිරි ලබාදුන්නේද? ඔව් ☐ නැත ☐

07. නැත්නම් ඒ සඳහා හේතු කවරේද?

1) මවගේ සංකූලතා ☐ 2) දරුවාගේ සංකූලතා ☐
3) දරුවාගේ කිරි ඉරීමේ ප්‍රශ්න ☐ 4) මව් කිරි ප්‍රමාණවත් නොවීම ☐

08. රෝහලෙන් පිටවීමට පෙර දරුවාට පිටි කිරි ලබා දුන්නේද? ඔව් ☐ නැත ☐

09. උපතෙන් පසු දරුවා හදිසි ප්‍රතිකාර අවශ්‍ය වූයේද? ඔව් ☐ නැත ☐

10. දරුවාගේ සෞඛ්‍ය තත්වය කෙසේද?

1) අඩුමාසයෙන් ඉපදීම ☐ 2) හෘදයේ රෝගී තත්ව ☐
3) දරුවා කහ වීම ☐ 4) විෂබීජ ඇතුල් වීම ☐
5) හුස්ම ගැනීමේ ප්‍රශ්න ☐ 6) දරුවාගේ සංජානීය ආබාධ ☐
7) උණ 100F ට වැඩි ☐ 8) මීකෝනියම් තිබීම ☐
9) දරුවා නිසි ආකාරව වර්ධන වී නැත ☐ 10) වෙනත් ☐

වාට්ටු අංකය :

අනු අංකය :

ඔබ විසින් පිළිතුරු සැපයීම සඳහා

උතුරු කොළඹ ශික්ෂණ රෝහලේ සිදුවන පෙර - සැලසුම් නොකර සිදු වු ගර්භනීභාවයන් ගණනය කිරීම, එයට අදාල කරුණු සහ දරු ප්‍රසුතියෙන් පසු ඇතිවන ප්‍රතිඵල සොයා බැලීමේ අධ්‍යයනය

පළමු සංස්කරණය 2017 ජුනි 29.

3 කොටස

අ - ගර්භනී භාවය ඇති වූ අවස්ථාව සම්බන්ධ තොරතුරු

පහත ප්‍රශ්න ඔබගේ ගර්භනී භාවය ඇතිවූ කාල සීමාවේ ඔබගේ සිතුවිලි හා අදහස් වල ස්වාභාවය විමසනු ලබයි. පිළිතුරු සැපයීමේදී ඔබගේ වඩාත් ආසන්නතම ගර්භනී භාවය සලකන්න.

01. මා ගර්භනී බව දැනගත් මුල් මාසයේදී (ඔබට වඩාත් අදාල පිළිතුර තොරන්න)

1) මම/අප දෙදෙනා උපත් පාලන ක්‍රමයක් භාවිත නොකලේය. ☐

2) මම/අප දෙදෙනා උපත් පාලන ක්‍රමයක් භාවිත කල නමත් සෑම විටකදීම භාවිතා නොකලේය. ☐

3) මම/අප දෙදෙනා උපත් පාලන ක්‍රමයක් සෑම විටම භාවිත කල නමුත් එම ක්‍රමවේදය නිවැරදි නොවීය. ☐

4) මම/අප දෙදෙනා උපත් පාලන ක්‍රමයක් සෑම විටම භාවිත කලේය. ☐

02. මව් පදවිය ලැබීම සඳහා මාගේ මෙම ගර්භනී භාවය ඇති වූයේ, (ඔබට වඩාත් අදාල පිළිතුර තොරන්න)

1) සුදුසු අවස්ථාවේය. ☐

2) සුදුසුය, නමුත් නිවැරදි වේලාවේ නොවේ. ☐

3) නුසුදුසු අවස්ථාවේ. ☐

03. මෙම ගර්භනී තත්වය ඇත විමට මද කාලයකට පෙර.(ඔබට වඩාත් අදාල පිළිතුර තොරන්න)

1) මම ගර්භනී වීමට සිතා සිටියෙමි. ☐

2) මගේ අදහස ස්ථාවර නැත (වෙනස් වන සුළුය) ☐

3) මම ගර්භනී වීමට සිතා සිටියේ නැත. ☐

04. මම ගර්භනී වීමට සුළු කාලයකට පෙර(ඔබට වඩාත් අදාල පිළිතුර තොරන්න)

1) මා දරුවෙකු බලාපොරොත්තු විය. ☐

2) දරුවකු සම්බන්ධ ස්ථිර තීරණයක් නොවීය. ☐

3) දරුවකු අවශ්‍ය වූයේ නැත. ☐

05. මම ගර්භනී වීමට පෙර.(ඔබට වඩාත් අදාල පිළිතුර තොරන්න)

1) මම හා සැමියා විසින් මෙම ගර්භනී භාවය තීරණය කර සිටියෙමු. ☐

2) අප දෙදෙනා දරුවන් පිළිබද සාකච්ඡා කර තිබූ නමුත් මෙම ගර්භනී භාවය තීරණය කර සිටියේ නැත. ☐

3) අප දෙදෙනා කිසි දිනක තවත් දරුවන් පිළිබඳ තීරණය කර තිබුනේ නැත. ☐

06. මා ගර්භනී වීමට පෙර සෞඛ්‍ය තත්වය වර්ධනය කරගැනීම සඳහා කුමන හෝ සූදානම් වීමක් තිබුනේද? (අදාල සියළුම ක්‍රම තෝරන්න).

1) ෆෝලික් අම්ලය ලබා ගතිමි. ☐ 2) දුම් පානය නැවතීම.

3) මධ්‍යසාර පානය නැවතීම. ☐ 4) සෞඛ්‍යාත්මක ආහාර ගැනීම.

5) වෛද්‍යවරයාගේ අවවාද පිළිපැදීම. ☐ 6) වෙනත්.

7) මම ඉහත කිසිවක් සිදු කලේ නැත. ☐
